# Tumor spheroids accelerate persistently invading cancer cells

**DOI:** 10.1038/s41598-022-18950-x

**Published:** 2022-08-29

**Authors:** Melanie Audoin, Maria Tangen Søgaard, Liselotte Jauffred

**Affiliations:** 1 The Niels Bohr Institute, University of Copenhagen, Blegdamsvej 17, DK-2100 Copenhagen, Denmark; 2 Present Address: DTU Health Tech, Denmark’s Technical University, Ørsteds Pl. 344, 108, 2800 Kgs. Lyngby, Denmark

**Keywords:** Biophysics, Cancer

## Abstract

Glioblastoma brain tumors form in the brain’s white matter and remain one of the most lethal cancers despite intensive therapy and surgery. The complex morphology of these tumors includes infiltrative growth and gain of cell motility. Therefore, various brain-mimetic model systems have been developed to investigate invasion dynamics. Despite this, exactly how gradients of cell density, chemical signals and metabolites influence individual cells’ migratory behavior remains elusive. Here we show that the gradient field induced by the spheroid—accelerates cells’ invasion of the extracellular matrix. We show that cells are pushed away from the spheroid along a radial gradient, as predicted by a biased persistent random walk. Thus, our results grasp in a simple model the complex behavior of metastasizing cells. We anticipate that this well-defined and quantitative assay could be instrumental in the development of new anti-cancer strategies.

## Introduction

Glioblastoma cancer is the most common type of primary, malignant brain tumor in adults. Its high mortality rate is accredited to its aggressive invasion of the surrounding healthy tissue. Prior to invasion, epithelial (tissue-like) cancer cells gain motility and leave the primary tumor to invade and ultimately form secondary tumors elsewhere in the organ (or the organism). This transformation from epithelial to mesenchymal (fibroblast-like) is necessary for glioblastoma cells to gain motility. Mesenchymal motility or *crawling*—is associated with elongated cell shapes, reinforcement of the intracellular actin network and strong interaction or modification of the local microenvironment^1,2^. Moreover, the mesenchymal phenotype has been linked to augmented therapy resistance^3^. Although glioblastoma cells are known to migrate via mesenchymal migration modes, amoeboid (leukocyte-like) migration has also been recorded; perhaps as a way to further confer therapy resistance^4^. Hence, cancer cells can move in different modes either individually or in a variety of configurations and can switch between them in response to their environment^5,6^.

The motility of cancer cells has been studied in various in vitro tumor models grown from cancer cell lines (or tumor tissues) in extracellular matrix (ECM) or hydrogel environments, which enable the investigation of spheroid invasion and migration in a natural, yet controlled manner^7,8^.

An often-used model for cancer cell motility in homogeneous ECM is the persistent random walk (PRW). However, previous studies have shown that the most effective direction of glioblastoma cell motility is outwards and away from the spheroid^9^ and that this activity is poorly described by the PRW^10^. Hence, we wondered whether such migrating behavior is intrinsic to the individual cells or whether it is imposed by some external gradient in the vicinity of the tumor spheroid? Therefore, we hypothesized that the spherically symmetric gradient—imposed by the geometry of the spheroid gives rise to a bias which drives cell invasion along the radial vector. That is, the cell trajectories would follow a biased PRW (BPRW). To investigate this hypothesis, we tracked glioblastoma (U87-MG) cancer cells embedded in 3D ECM migrating either (i) individually or (ii) from multicellular spheroids. These experiments were complemented by simulation of trajectories in both assays. Although this phenomenon may not be observed across cancer types, we confirmed that indeed glioblastoma spheroids exert a repulsive gradient force which pushes the cells to migrate faster, straighter, and radially away from their origin, as predicted by a BPRW.

**Figure 1:**
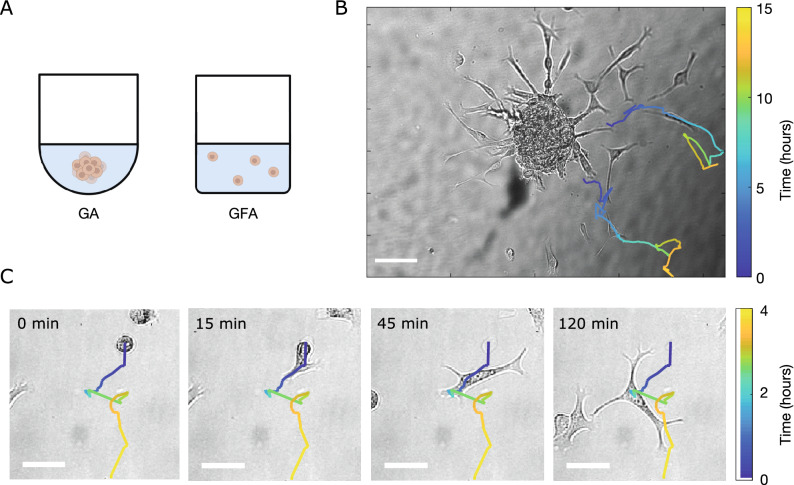
Brain cancer cells invading the ECM. (**A**) Schematics of an ECM-embedded spheroid in a gradient assay (GA) or individual cells in a gradient-free assay (GFA). (**B**) Tracks of migrating cells in a GA from $t={9} \,\hbox {h}$ and $t={5} \,\hbox {h}$, respectively, superimposed on the corresponding bright field image. For each track, the color bar indicates time evolution. Scale bar is ${100}\,\upmu \hbox {m}$. (**C**) Morphological transition of a cell prior to migration. Image sequence of brain cancer cells invading the ECM at $t=\{0,15,45,120\}$ min in GFA. The track of the cell is superimposed on the bright field image. The color bar indicates time evolution and the scale bars are ${50}\,\upmu \hbox {m}$.

## Results

Through precise cell tracking of human glioblastoma cells (U87-MG), we characterized the motility patterns of cells in 3D ECM consisting of 65% Matrigel$^{\mathrm{TM}}$  in cell culture medium. It has previously been shown that the trajectories of invading cells are independent of the concentration of the ECM, both in respect to directionality, speed, and persistence^9^. So, with the aim of comparing the metastatic spread of cells from tumor spheroids to the migration of individual cells, we prepared two assays as sketched in Fig. 1A.

First, spheroids were formed as a result of gravity-assisted accumulation of cancer cells at the bottom of U-bottom wells. After harvesting, the spheroids were embedded in ECM and incubated under physiologically relevant conditions. Then after a few hours, multicellular strands invaded the surrounding matrix and cells gradually left these strands to migrate individually. Representative snapshots of cells superimposed with trajectories are shown in Fig. 1B, where cells appear to move radially outward. Due to the spheroid-induced gradient, we refer to this as a gradient assay (GA).

In parallel, a gradient-free assay (GFA) was prepared by embedding non-spheroidal cell cultures in 65% Matrigel$^{\mathrm{TM}}$. Figure 1C shows representative bright-field images of an epithelial-to-mesenchymal transition of a single cell overlaid with the full cell trajectory. The cells were still round-shaped and immobile when first embedded in Matrigel$^{\mathrm{TM}}$  at $t=0$ min. However, with time (1–3 h) they switched to more elongated shapes with long lamellipodia—characteristic of mesenchymal motility^4^ and started migration.

**Figure 2:**
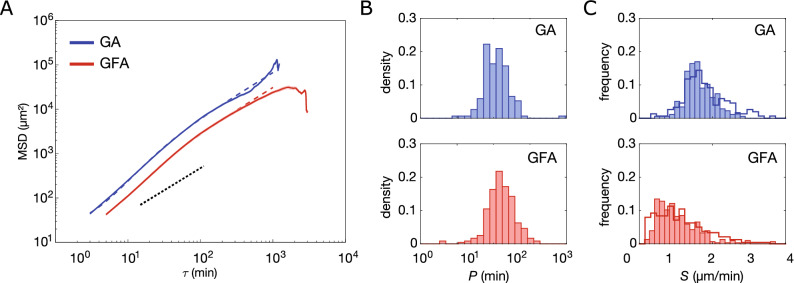
Ensemble-averaged mean-squared displacement analysis. (**A**) GA (N=177) and GFA (N = 305) $\text {MSD}(\tau )$ (mean ± SEM) on double-logarithmic scale. Dashed lines correspond to fitting of Eq. 2 ($R^2>0.99$). Black punctuated line has a slope of 1, which corresponds to a diffusive motion and is inserted as a guide to the eye. (**B**) Distributions of persistence times, *P* on a log-normal scale, of the cells in GA ($N=156$ with $R^2>0.99$) and in GFA ($N=251$ with $R^2>0.99$ ). Values are obtained from the msd-analysis described in Eq. (2). (**C**) Distributions of migration speed, *S*, for GA and GFA, both fitted from Fürth’s formula (full line) as in (**B**) and from averaging over the individual trajectories (bars) calculated from Eq. (4).

### Cells migrate with persistence

From the set of trajectories, we calculated the ensemble-averaged distance that the cells move in a given time interval, $\tau$, which is designated as the lag or delay time^11^:

1 $$\begin{aligned} {\displaystyle \mathrm{{MSD}}\equiv \langle |{\mathbf {x}} (\tau )-{\mathbf {x}}(0) |^{2}\rangle }, \end{aligned}$$

where $\langle \ldots \rangle$ signifies an ensemble average. MSD curves for GA and GFA are shown in Fig. 2A, where the black punctuated line (slope of 1) corresponds to freely diffusing cells i.e. Brownian motion. Hence, in both cases cells seems to be super-diffusing (slope $>1$) in accordance with prior findings^9,12^.

Metastatic cancer cells-like many other motile cells—actively move with persistence. This behavior is often modeled by a random walk (RW) with memory, termed a persistent random walk (PRW). A cell moving with persistence is more likely to keep moving in the same direction than in any other. Therefore, any given ensemble of trajectories can be identified by a characteristic time termed the persistence time, $\langle P\rangle$, where again $\langle \ldots \rangle$ signifies the ensemble average. Fürth’s formula for a 3D cell trajectory imaged in 2D is:

2 $$\begin{aligned} \text {MSD}(\tau )=2\langle S\rangle ^2\langle P\rangle ^2\left( e^{-\frac{\tau }{\langle P\rangle }}+\frac{\tau }{\langle P\rangle }-1 \right) \end{aligned}$$

where $\langle S\rangle$ is the average speed over the entire ensemble of trajectories and $\tau$ is still the time delay. The fits are inserted in Fig. 2A (dashed lines) for both GA (blue) and GFA (red).

At long delays $\tau>>\langle P\rangle$, the PRW model predicts that the MSD follows a linear regime $MSD\propto \tau$ (punctuated black line). This is observed in Fig. 2A in which the slopes of the ensemble-averaged MSDs decrease for $\tau >{200}$ min proving that the cells lose their super-diffusivity over time. However, when $\langle P\rangle$ is of the order of $\tau$ (see Fig. 2A) the cells exhibit anomalous diffusivity, as earlier reported^9,13^. This regime change might be due to cell-cell adhesion or pauses during mitosis. Additionally, the mesh size of the extracellular matrix is not perfectly homogeneous which is likely to influence cell motility and accordingly the persistence of the motion. Moreover, the MSD values for $\tau >800$ min suffer from large uncertainties due to the sparse number of data points. Hence, we cannot conclude on these apparent drastic changes at long delays.

Any individual trajectory in the ensemble can also be identified by a characteristic *P*, such that the time-averaged mean-squared displacement is

3 $$\begin{aligned} \text {msd}(\tau )=2S^2P^2\left( e^{-\frac{\tau }{P}}+\frac{\tau }{P}-1 \right) , \end{aligned}$$

where *S* is the average speed over the cell’s trajectory. We therefore fitted all individual GA (blue) and GFA (red) cell trajectories’ msd (Eq. 11) and found the distributions of *P* and *S* shown in Fig. 2B,C, respectively. The *P*-distributions are log-normal and similar, with most $P<90$ min: For GA $\langle P\rangle =(12.7\pm 1.0)$ min (mean+SD) of logarithmic values, and for GFA $\langle P\rangle =(16.6\pm 1.0)$ min of logarithmic values. We also observe a few highly persistent cells in GA as earlier reported^9^. As these highly persistent cells are only found escaping spheroids (GA), we suggest that this is more likely to be related to local chemical gradients or remodeling of the extracellular matrix rather than genotype or other cell-intrinsic factors. The PRW model assumes that cells migrate with a constant persistence and we thus estimate an average *P* for the entire trajectory even if Fig. 2 indicates that cell motility modes change at long time-scales^14^.

The effect of the spheroid on cell motion can be further characterized by the distributions of cell speed, *S*, shown in Fig. 2C, which were obtained in two ways: either through msd-fitting with Eq. 2 (line) or by averaging over the individual trajectories (bars) as shown below

4 $$\begin{aligned} S=\vert \langle {\mathbf {v}}(t_i)\rangle _t\vert , \end{aligned}$$

where $\langle \ldots \rangle _t$ indicates the average over all $t_i$’s of a trajectory, where $i\in \{1,2,3\ldots \}$ is the index of discrete time steps. The distributions of *S* in Fig. 2C exhibit a noticeable right-skew in the GFA compared to GA. Moreover, a Kolmogorov-Smirnov test rejects the null hypothesis that the *S*-distributions are normal distributions.

From fits of Eq. 3, we found that for cells in GA, $\langle S\rangle =(1.81\pm 0.66)\,\upmu \hbox {m}/\hbox {min}$ (mean+SD) and in GFA, $\langle S\rangle =(1.15\pm 0.71)\,\upmu \hbox {m}/\hbox {min}$ (summarized in Table 1). So, to determine *S* and *P* we found the mean of the time-averaged msd (Eq. 11) rather than from the fit of Eq. 2 shown in Fig. 2A), which would yield slightly different values, see Ref.^15^ for a discussion and an overview. Nevertheless, the spheroid seems to induce a repulsion that drives cells persistently and fast away from it.

### Cells do occasional U-turns

The angular displacement is defined as the magnitude of the angle, $\theta (\tau )$, between two velocity vectors separated by the delay $\tau$, i.e., between ${\mathbf {v}}(t_i)$ and ${\mathbf {v}}(t_i+\tau )$:

5 $$\begin{aligned} \theta (\tau )=\cos ^{-1}\left( \frac{{\mathbf {v}}(t_i) \cdot {\mathbf {v}}(t_i+\tau )}{\vert {\mathbf {v}}(t_i)\vert \vert {\mathbf {v}}(t_i+\tau )\vert }\right) . \end{aligned}$$

The distributions of $\theta (\tau )$ in Supplementary Fig. S1 show that cells ($\tau =15$ min) will most often continue in the same or a very similar direction (small $\theta$) for both the GA and GFA. This behavior reveals that for short delays, successive velocity vectors are highly correlated. However, for longer $\tau$ the distributions flatten which indicates that the cell motion gets increasingly uncorrelated with time.

It is worth noticing that for all $\tau$’s, $\theta$ peaks at $0^{\circ }$ and $180^{\circ }$. This indicates an anisotropy in the cell migratory path and proves the existence of an axis along which the cells preferably migrate forward and backwards. As mesenchymal cells proteolytically degrade the matrix when moving through it^16–18^, they will most probably use already degraded ECM tunnels when available—either straight ahead or by making a U-turn as found earlier for human fibrosarcoma in agarose (GFA)^13^ and for U87-MG in a similar assay (GA)^9^.

### Cells leave spheroids by moving fast along a radial gradient

**Figure 3:**
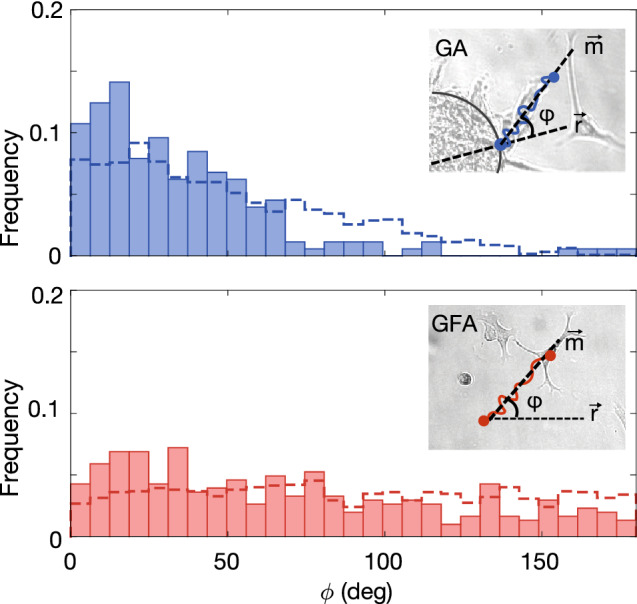
Distribution of angle, $\varphi$, between the major axis, ${\mathbf {m}}$, and initial radial direction, ${\mathbf {r}}$, for data (bars) and simulation (dashed line) from GA (N = 177) and GFA (N = 305). Insets: Schematic of the angle $\varphi$ between major direction of motion, ${\mathbf {m}}$, and initial radial direction, ${\mathbf {r}}$ for GA and GFA, respectively.

As our goal was to capture the differences between the two assays, we developed a set of descriptive and fairly simple models to complement our experimental data. We used a continuous stochastic model to simulate cell trajectories, where a cell was represented as a single point corresponding to its center-of-mass and its 2D projected trajectory ${\mathbf {x}}(t)$, which consisted of *n* steps of size *dt*. In particular, we focused on a PRW model as cell migration has been reported to be well described by this^13^. We hypothesized that the gradients imposed by the spheroid would result in an apparent bias in the cell trajectories i.e. following a BPRW model, as previously suggested^19^. Specifically, we anticipated this bias to be a repulsive field in the direction of the spheroid and decaying as $1/|{\mathbf {r}}|$, where ${\mathbf {r}}$ is the radial vector. The model is described in details in the “Materials and methods” section and parameter values are summarized in Table 1.

We thus turned to a quantitative description of the differences observed in Fig. 1, in particular regarding the direction of motion. To do so, we defined the following two vectors: the major axis of motion, ${\mathbf {m}}$, and the radial vector, ${\mathbf {r}}$. The first is

6 $$\begin{aligned} {\mathbf {m}}= \langle {\mathbf {x}}(t_i)-{\mathbf {x}}(t_i-1)\rangle _t, \end{aligned}$$

where $\langle ..\rangle _t$ signifies the average displacement vector of the trajectory, i.e., averaged over all $t_i$’s. The radial vector is

7 $$\begin{aligned} {\mathbf {r}} = {\left\{ \begin{array}{ll} {\mathbf {x}}(0) &{} \text{ for } \text{ GA } \\ {\mathbf {x}}(1)-{\mathbf {x}}(0) &{} \text{ for } \text{ GFA }, \end{array}\right. } \end{aligned}$$

where the position vector ${\mathbf {x}}(0)$ equals the radial vector from the center of the spheroid, (0, 0), to the initial position on the spheroid surface, as sketched in the inset of Fig. 3 (GA). As GFA lacks radial symmetry, we defined the radial vector, ${\mathbf {r}}$, in this assay to be the displacement vector of the first step of the trajectory (see inset of Fig. 3). Given this, $\varphi$ is the angle between the vectors ${\mathbf {m}}$ and ${\mathbf {r}}$. The $\varphi$-distributions for all trajectories in GA (blue) and GFA (red) are given in Fig. 3, where the dashed lines correspond to the distributions of simulated trajectories. To a great extent, cells in the GA environment follow the radial vector away from the spheroid ($\varphi <75^{\circ }$). In contrast, cells in the GFA do not migrate along any predefined direction which renders the $\varphi$’s evenly distributed. Furthermore, this is reflected in the medians, which are $35^{\circ }$ and $71^{\circ }$ for the GA and GFA cells, respectively. Hence, these results suggest that the spheroid repulses cells in the radial direction specifically.

### Cells migrate with high directionality away from spheroids

**Figure 4:**
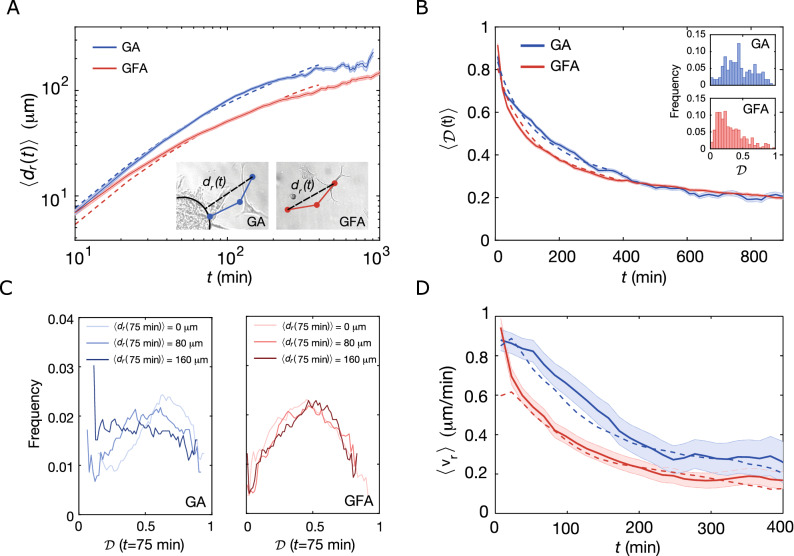
Time evolution of migration patterns. (**A**) Time-dependent ensemble-averaged radial displacement, $\langle d_r(t_i)\rangle$, versus time, *t*, (mean ± SEM) for GA (N=177) and GFA (N = 305) on a double-logarithmic scale. Dashed lines correspond to results obtained from BPRW (blue) and PRW (red) simulations. Insets: Sketches defining the radial displacement, $d_r(t_i)$, for GA and GFA, respectively. (**B**) Time evolution of the ensemble-averaged directionality, $\langle {\mathscr {D}}(t_i)\rangle$ (mean ± SEM) for GA (N = 177) and GFA (N = 305). The dashed lines are averages over simulated tracks for BPRW (blue) and PRW (red). Insets: Distribution of the directionality of the entire trajectories, ${\mathscr {D}}$. (**C**) Distribution of directionalities of the trajectories after $t=75$ min, ${\mathscr {D}}({75}\,\hbox{min})$, at different distances from initial position, $\langle d_r({75}\,\hbox {min})\rangle$, for GA (blue) and GFA (red). (**D**) Time-evolution of the ensemble-averaged radial velocity, $\langle v_{r}(t_i)\rangle$, (mean ± SEM) for GA and GFA and simulated trajectories (dashed lines) from BPRW (blue) and PRW (red). In all four cases, $\langle v_{r}(t_i)\rangle$ was binned over 15 min with a moving average of 10 data points.

To investigate this further, we calculated the time evolution of the radial distance traveled by the cells:

8 $$\begin{aligned} d_r(t_i) = {\left\{ \begin{array}{ll} |{\mathbf {x}}(t_i)|-|{\mathbf {x}}(0)| &{}\text{ for } \text{ GA } \\ |{\mathbf {x}}(t_i)-{\mathbf {x}}(0)| &{}\text{ for } \text{ GFA}, \end{array}\right. } \end{aligned}$$

as sketched for both assays in the insets of Fig. 4A. The time evolution of the ensemble-averaged radial displacement, $\langle d_r(t_i)\rangle$, is also shown in Fig. 4A. At any given time point, the GA cells have on average migrated further from their starting point than cells in the GFA. This is in agreement with the observation that GA cells migrate faster than cells in the GFA (Fig. 2). It is further justified by the $\langle d_r(t_i)\rangle$ obtained from simulated trajectories of both BPRW (blue dashed line) and PRW (red dashed line). To understand this behavior further, we show the radial distance (eq. 8) of individual trajectories in Supplementary Fig. S2 and find that he smooth ensemble averages of $d_r(t_i)$ covers very noisy individual trajectories.

We find that the distance traveled by a cell also depends on the time-dependent directionality, ${\mathscr {D}}(t_i)$. It is defined as the ratio between a cell’s distance from its initial position, ${\mathbf {x}}(0)$, and the trajectory length at time $t_i$:

9 $$\begin{aligned} {\mathscr {D}}(t_i) = \frac{\vert {\mathbf {x}}(t_i)-{\mathbf {x}}(0)\vert }{\sum ^{t'=t}_{t'=1}\vert {\mathbf {x}}(t')-{\mathbf {x}}(t'-1)\vert }. \end{aligned}$$

So by definition $0\le {\mathscr {D}}\le 1$, where ${\mathscr {D}}=1$ corresponds to a ballistic trajectory and ${\mathscr {D}}=0$ is an infinitely long and tortuous random walk. Figure 4B shows the ensemble-averaged directionality, $\langle {\mathscr {D}}(t_i)\rangle$, for both GA (blue) and GFA (red) cells as well as for the simulated trajectories (dashed lines). For $t<400$ min, cells in GA exhibit higher directionality than GFA cells, which proves that cells in GA indeed move straighter. However, at longer time scales, the GA $\langle {\mathscr {D}}(t_i)\rangle$ falls off to values closely matching those of cells in GFA. This confirms the idea of a repulsive field around the spheroid with a gradient of $-1/{\mathbf {r}}^2$. It follows that once the GA cells escape this field, they slow down and their migration patterns start to resemble those of cells moving in a gradient-free environment. The insets in Fig. 4B show distributions of directionalities for the entire trajectories, ${\mathscr {D}}$. As expected and despite large variations cells in GA move with higher overall directionality than cells in GFA.

To test the prediction that the bias field is dependent on the distance from the spheroid center, (0, 0), we examined how distributions of ${\mathscr {D}}$ changed at $t=75$ min with $d_r({75}\,\hbox {min})=\{ 0,80,160 \}\,\upmu \hbox {m}$ as shown in Fig. 4C. For GA, the distributions shift towards lower ${\mathscr {D}}$ as $d_r(t_i)$ increases, in accordance with the idea that the gradient is strongest close to the spheroid ($d_r({75}\,\hbox {min})=0$ $\upmu$m vs. $d_r({75}\,\hbox {min})={160}\,\upmu \hbox {m}$). In contrast for cells migrating in GFA, there are no spatial changes of $d_r(t_i)=\{ 0,80,160 \}$ $\upmu$m as all of them overlap. These results suggest that in the vicinity of the spheroid, cells migrate more efficiently through the ECM. On the other hand, in GFA cell motion is more diffusive so the cells explore more thoroughly their local environment. This means that the cells’ migration strategy is highly environmentally dependent and is balanced between local exploration and a more directed migration mode.

### Spheroids accelerate the radial velocity

We have now established that metastatic glioblastoma spheroid cells invade their surrounding environment faster, more persistently, and straighter than single cells in the exact same ECM. However, a potential time and space dependence of the proposed bias still needs to be investigated. As the gradient is anti-parallel with the radial vector ${\mathbf {r}}$, we measured the time and space dependence of the radial velocity i.e. the scalar projection of ${\mathbf {v}}(t)$ onto the unit radial vector:

10 $$\begin{aligned} v_r(t_i)={\mathbf {v}}(t_i)\cdot \frac{{\mathbf {r}}}{\vert {\mathbf {r}}\vert }. \end{aligned}$$

In the GA, the ensemble-averaged radial velocity, $\langle v_r(t_i)\rangle$, decays with respect to the radial distance, $d_r(t_i)$, for the first $\sim {100}\,\upmu \hbox {m}$ after leaving the spheroid (Supplementary Fig. S3) after which the spheroid-induced field becomes negligible and the velocity reaches $\langle v_r(t_i)\rangle \sim {0.4}\,\upmu \hbox {m}/\hbox {min}$. This decay in velocity is, thus, a manifestation of the space dependence of the bias induced by the spheroid. In contrast, in GFA there is no such dependence. Thus, cells in this assay migrate with constant $\langle v_r(t_i)\rangle \sim {0.2}\,\upmu \hbox {m}/\hbox {min}$ through a seemingly isotropic space.

Furthermore, $\langle v_r(t_i)\rangle$ decays with time for both GA and GFA as shown in Fig. 4D. However, for the first part ($t_i<200$ min) the radial velocity of GA is significantly larger than in the GFA. The agreement between experiments and simulations confirms that the spheroid-induced field is well described by a $-1/\vert {\mathbf {r}}^2\vert$ gradient and the bias term of Eq. 17.

## Discussion and conclusion

Here, we report migration patterns of glioblastoma U87-MG cells in brain-mimetic culture models. We showed that cells fleeing a spheroid tumor model (GA) move with persistence, much like single cells in an analogous gradient-free microenvironment (GFA). More interestingly, we found that cells are accelerated along the outward-pointing gradient of a spheroid and that this results in faster and more directed migration patterns.

Our results are consistent with persistent motion, as U87-MG cells tend to have aligned velocities at short time scales which means they follow a preferred direction of motion with persistence; given by $P>0$. However at long time lags, motion is uncorrelated (i.e. persistence is lost) and cells move diffusely. This may arise from the collisions and changes in the cells’ environment such as chemical cues and extracellular matrix that is being constantly remodeled. Therefore, the persistence observed for cells in the GFA is captured by the PRW model, which describes the motion of a self-propelled cell migrating along a preferred axis. However, this model fails to describe the gradient force exerted by the spheroid. We showed that the induced bias drives the cells’ motion along the radial axis and increases their diffusivity. Assuming that the spheroid only has an external influence on cell migration i.e. its presence changes the external environment but has no intrinsic effect on the cell the effect of the spheroid can be modeled by adding an external bias term in the Ornstein-Uhlenbeck process. The resulting model is a biased PRW (BPRW) model whose bias amplitude decreases with the distance from the spheroid^19^. Due to the simplicity of the model system and lack of an explicit external attractant, we suggest the existence of a repulsive force that drives cells away from the spheroid. This driving force arises as a result of spheroid formation which among other things increases cell density. It is as such system-intrinsic and its origin could include gradients in e.g. matrix composition and stiffness^20,21^, pH^22^, oxygen^23^, secreted factors^24^ or nutrients found in the Matrigel. Of note, others have found that melanoma and other cell types are able to self-generate such gradients^25,26^. Irrespective of the origin, it can be debated whether the cells are propelled forward by the stiff/acidic/hypoxic environment in the spheroid or attracted by the less stiff/neutral pH/oxygenated/nutrient dense environment surrounding the spheroid. In this respect, our model can be modified accordingly to describe attraction of the cells towards an external attractant with only minor differences in the obtained migration speed and bias responsiveness. Apart from these attractants/repellents the reorganization of the matrix network as the cells move through it has also been found to enhance velocity^16–18^. The nature of such cell-matrix reorganization includes irreversible proteolysis^27,28^. We also find indications of this behavior, however, the loss of anisotropy over time suggests that the open paths could be closing again for large time delays. Therefore despite the cells’ persistence, the close interplay with the local environment will gradually stray the cells from their primary directions of motion. Although the anisotropic PRW (APRW) model has proven to be an accurate description of 3D cancer cell migration in gradient-free environments^13^, we chose to use the simpler PRW model which still captures the most important features of cell migration—as the basis for our BPRW model. Nevertheless, the non-normal cell speeds we found taken together with the outliers in the persistence times indicate that the ensemble of cells includes sub-populations of particularly fast cells; as recently proposed^9,10^. Therefore, more data is needed to put new models forward which include population heterogeneity.

Even though model systems like matrix-embedded spheroids have greatly helped our understanding of cancer metastasis, they cannot recreate the entire metastatic process. Therefore, we limited our study to the initial invasion of the local microenvironment during which we identified a strong and space-dependent repulsive force on migrating cells caused by the cancer tumor. Furthermore, spheroids embedded in an artificial matrix only provide a very simplified model of cancer and do not completely recapitulate the native brain environment which includes e.g. vasculature, signaling molecules as well as immune and other cell types^29^. The validity of our in vitro findings could be extended by studying the migration of human glioma xenografts^30^ or multi-cell type glioblastoma spheroids^31^ embedded in ex vivo organotypic brain slices. In addition, cranial window models provide a promising opportunity for studying cell migration in vivo^32^. Therefore, further experiments should be conducted to estimate the cues and identify more specifically the nature and chemical origin(s) of the repulsion. One possible direction would be to mimic gradients of cell density or chemical factors as was previously done for breast cancer cells in a growth factor gradient^33^.

Temozolomide (TMZ) treatment has been shown to reduce U87-MG spheroid invasion by 80%^34^, however, TMZ-treated spheroids show a higher viability than individual cells in suspension^35^. It is thus likely that TMZ will also differentially affect the migratory behavior of spheroids (GA) vs. individual cells (GFA). The assay we reported here could be used to assess the effect of TMZ and similar drugs on glioblastoma cell migration. This will help further characterize in a quantitative manner the effect of TMZ on cell migration behavior and improve therapeutic efficiency. For instance, by targeting the cells with the strongest drug sensitivity or by assessing the overall effect of the drug on both individual and spheroid cells that are involved in the invasion process. Hence this approach is also promising for large drug-screening processes^36,37^ and development of new anti-cancer strategies.

## Materials and methods

### Cell culture

Uppsala 87 Malignant Glioma (U87-MG)^38^ cell line was a kind gift of Prof. Petra Hamerlik (Danish Cancer Society’s Research Center, Copenhagen, Denmark). Cells were cultured in Dulbecco’s Modified Eagle Medium (DMEM) with 10% Fetal Bovine Serum (FBS) and 1% penicillin/streptomycin (Gibco) at $37\,^{\circ }\hbox {C}$ and 5% $\hbox {CO}_2$ and harvested at 90% confluency.

### Single cell migration assay: GFA

In ultra-low attachment 96-well flat-bottom microtiter plates (Corning), $\sim 3500$ cells pr. well were embedded in a final volume of $100\,\upmu \hbox {L}$ of 65% GFR Membrane Matrix Matrigel$^{\mathrm{TM}}$ (Fischer Scientific) mixture in medium. Prior to mixing, Matrigel$^{\mathrm{TM}}$ was thawed on ice (or overnight at $4\,^{\circ }\hbox {C}$) and pipette tips were stored at $-20\,^{\circ }\hbox {C}$. Furthermore, all experiments in this study were performed using the same batch of Matrigel$^{\mathrm{TM}}$  to account for natural variations. After mixing cell culture and Matrigel$^{\mathrm{TM}}$ mixture, the plate was incubated ($37\,^{\circ }\hbox {C}$ and 5% $\hbox {CO}_2$) for $\sim$1 h until it had solidified completely. Then ${100}\,\upmu \hbox {L}$ medium was added to each well to ensure plentiful nutrient supplies and avoid condensation on the inside of the plate cover when imaging. We performed 2 independent experiments and collected data from 8 different wells, which after analysis resulted in a total of 305 cell trajectories.

### Spheroid invasion assay: GA

Tumor spheroids were formed by suspending $\sim 325$ cells in ${200}\,\upmu \hbox {L}$ cell culture medium pr. well in a 96-well ultra-low attachment U-bottom plate (Corning) and incubated ($37\,^{\circ }\hbox {C}$ and 5% $\hbox {CO}_2$) for $\sim 72$ h. At this point, spheroids had reached a diameter of about $\sim {80}\,\upmu \hbox {m}$. Then the spheroids were embedded in 65% Matrigel$^{\mathrm{TM}}$  by aspirating ${120}\,\upmu \hbox {L}$ medium and adding ${150}\,\upmu \hbox {L}$ ice cold Matrigel$^{\mathrm{TM}}$  as detailed in^9,39^. The plates were incubated ($37\,^{\circ }\hbox {C}$ and 5% $\hbox {CO}_2$) to solidify completely ($\sim$1 h) before adding ${100}\,\upmu \hbox {L}$ cell culture medium pr. well. We performed 2 independent experiments with 4 and 5 technical replicates, which after analysis resulted in a total of 177 cell trajectories.

### Imaging

The Matrigel$^{\mathrm{TM}}$ -embedded cells and spheroids in flat-bottomed or U-bottom 96-well plates were time-lapse-imaged using a JuLI$^{\mathrm{TM}}$  stage real-time cell history recorder (NanoEntek, South Korea) placed inside an incubator ($37\,^{\circ }\hbox {C}$ and 5% $\hbox {CO}_2$). Using the built-in software, exposure time, brightness, and focus were adjusted for each well. Time-lapse bright-field images were obtained using the fully automated x-y-z stage, every 3 min (GA) or 5 min (GFA) for at least 24 h using a 10x objective to obtain a resolution of ${440}\,\upmu \hbox {m/pixel}$. The recorded images are 2D projections of a 3D migration. If a cell migrates too far away from the focal plane it is not visible anymore. The same goes if the cell migrates outside the field of view^13,33^.

### Image analysis

To aid image analysis, we obtained binary masks of the raw images using the machine-learning-based detection algorithm Ilastik^40^. These masks were used as input for the TrackMate plugin^41^ for Fiji^42^ which extracted the trajectory, [*x*(*t*), *y*(*t*)], for each cell’s center of mass in a given time-lapse. We subsequently performed manual editing of the tracks with TrackScheme, as TrackMate might falsely detect or miss a few number of steps or links. Following the criteria discussed in ref.^43^, we terminated tracks when cells had migrated either (i) along the edge of the image for more than 15 min, (ii) less than 50 $\upmu$m over their entire trajectory to remove the contribution from immobile/dead cells, as well as when cells either (iii) divided or (iv) merged. The quality and reliability of cell motility analysis is highly dependent on the image acquisition procedures. As we used a motorized stage during image acquisition, the vibrations or drift of that stage will translate into apparent cell movements. Therefore, we used plastic beads as phantom cells in ECM to evaluate the vibration noise. We concluded that the stage vibration is negligible, so the measured cell trajectories were directly used for the analysis without correction.

### Mean-squared displacement fitting

In order to characterize migration, we measured the diffusion via the time-averaged squared distance (msd) traveled by a cell over some time lag, $\tau$:

11 $$\begin{aligned} \text {msd}(\tau ) =\langle [{\mathbf {x}}(t_i+\tau )-{\mathbf {x}}(t_i)]^2\rangle _t, \end{aligned}$$

where ${\mathbf {x}}(t_i)$ is the time-dependent position vector at the *i*-th time step, $t_i$, and $\langle \ldots \rangle _t$ indicates an average over the entire cell trajectory.

We fitted the msd with Eq. 2 to obtain the corresponding set of persistence time, *P*, and average speed, *S*, for each cell. Expectation values were obtained by fitting the resulting distributions (GFA, GA) with a log-Gaussian (Fig. 2B) or Gaussian (Fig. 2C), respectively.

We also found the ensemble averaged msd, MSD, and because these fits use non-linear least square methods, the results are highly dependent on the weighting function which compensates for the loss of resolution in the computation at large $\tau$^44^:

$$\begin{aligned} W(a)=\text {MSD}(\tau ) \left( \frac{(2a^2+1)}{3a(A-a+1)}\right) ^2, \end{aligned}$$

where $\tau$ is the time lag, as in Eq. 11 and $a=\{1,2,3,\ldots ,A\}$ is the number of points used for the fit, where we chose *A* to be $25\%$ of the trajectory length to optimize the fits and discarded all fits with $R^2 < 0.99$, as detailed in^9^ and references therein.

### Trajectory simulations

The BPRW simulation is described by a normalized Langevin equation, which provides the velocity change between two successive steps $d\tilde{{\mathbf {v}}}$. For the BPRW, the Langevin is the sum of the resistance to motion (first term), a time-dependent bias (second term), and random fluctuations (third term)^45^:

12 $$\begin{aligned} d\tilde{{\mathbf {v}}}(t_i) = (1-d\tilde{t_i})\, d\tilde{{\mathbf {x}}}(t_i-1) + \Psi (t_i)d\tilde{t_i} + \sqrt{d\tilde{t_i}}\,{\tilde{N}}(0,1), \end{aligned}$$

where $\tilde{{\mathbf {x}}}(t_i)$ is the normalized position vector, $d\tilde{t_i}$ is the normalized time step, $\Psi (t_i)$ the bias induced by the spheroid and ${\tilde{N}}(0,1)$ a scalar drawn from the standard normal distribution.

In order to get the normalized Langevin (Eq. 12), the position vector is normalized as

13 $$\begin{aligned} \tilde{{\mathbf {x}}}(t_i)=\frac{{\mathbf {x}}(t_i)}{\langle P\rangle \langle S\rangle }, \end{aligned}$$

where the mean persistence time, $\langle P\rangle$, and migration speed, $\langle S\rangle$, are averaged over all experimentally-obtained cell trajectories for either the spheroid invasion assay (GA) or the single-cell migration assay (GFA); as detailed above.

The time step is re-scaled as:

14 $$\begin{aligned} d{\tilde{t}}=\frac{dt}{\langle P\rangle } \end{aligned}$$

and the resulting trajectories have steps of

15 $$\begin{aligned} d\tilde{{\mathbf {x}}}(t_i)=d\tilde{{\mathbf {v}}}(t_i)d{\tilde{t}}, \end{aligned}$$

such that the position vector at time *t* becomes

16 $$\begin{aligned} \tilde{{\mathbf {x}}}(t_i)=\tilde{{\mathbf {x}}}(t_i-dt)+d\tilde{{\mathbf {v}}}(t_i)d{\tilde{t}}. \end{aligned}$$

In the homogeneous environment experienced by migrating cells in the GFA, we assume that the bias term is null ($\Psi =0$) and the model in Eq. 12 reduces to a PRW model. In contrast in the GA case, we assume that the spheroid induces a bias ($\Psi \ne 0$). This bias is presumably repulsive, radial and negligible far from the spheroid. Therefore, we hypothesize that the space-dependent field is proportional to $-1/\vert {\mathbf {r}}\vert$, where ${\mathbf {r}}$ is the radial vector pointing outwards from the spheroid’s center to the cell position. We also assume the bias to be repulsive i.e the gradient is largest when $d{\mathbf {x}}(t_i-dt)$ is anti-parallel to ${\mathbf {r}}(t_i)$. The resulting time-dependent drift is therefore described by:

17 $$\begin{aligned} \Psi (t_i)=\frac{\delta {\tilde{R}}}{\vert {\mathbf {r}}(t_i-dt)\vert ^2}\,\sin \Big |\frac{\rho (t_i)}{2}\Big |\,\mathbf {\frac{{\mathbf {r}}}{\vert {\mathbf {r}}\vert }}, \end{aligned}$$

where $\delta$ is a dimensionless responsiveness to the bias, ${\mathbf {r}}/\vert {\mathbf {r}}\vert$ is the unit radial vector, ${\tilde{R}}$ is the re-scaled radius of the spheroid, $R_0$, such that

18 $$\begin{aligned} {\tilde{R}}=\frac{R_0}{\langle P\rangle \langle S\rangle } \end{aligned}$$

and $\rho (t_i)$ is the angle between ${\mathbf {r}}(t_i)$ and the displacement vector, $d{\mathbf {x}}(t_i-dt)$:

19 $$\begin{aligned} \rho (t_i) = \cos ^{-1}\left( {\frac{{\mathbf {r}}\cdot d{\mathbf {x}}(t_i-dt)}{\vert {\mathbf {r}}\vert \vert d{{\mathbf {x}}}(t_i-dt)\vert }}\right) . \end{aligned}$$

Both the direction and amplitude of the bias were inferred from the experimental results, as well as $\langle P\rangle$ and $\langle S\rangle$. Hence, $\delta$ was found by comparing the ensemble-averaged radial velocity in Eq. (10) of data with the ensemble-average of $N=1000$ simulations of trajectories described by Eqs. (13–19), with $dt=0.5$ min, $n=800$ steps and a minimal displacement, $d={50}\,\upmu \hbox {m}$ to remove the contribution from immobile/dead cells. Then, by minimizing the normalized root-mean-squared error we estimated the bias amplitude to be $\delta =3$. Taking all this together, trajectories for a BPRW and PRW were obtained with the equations above, with N = 1500, $n=800$, $dt={0.5}\,\hbox {min}$ and the following model parameters:

**Table 1:** Simulation parameters.

	$\delta$	*P* (min)	$S\,(\upmu \hbox {m/min})$
BPRW	3	12.7	1.81
PRW	0	16.6	1.15

## Supplementary Information

Supplementary Figures.

## Data Availability

All cell trajectories from data as well as from simulations that are presented in this study are openly available at FigShare at 10.6084/m9.figshare.19418963.
