# Supplementary material for: Tumor spheroids accelerate persistently invading cancer cells
Source: Sci Rep. 2022 Aug 29;12:14713. doi: 10.1038/s41598-022-18950-x (PMC9424244; doi:10.1038/s41598-022-18950-x)
Supplement: Supplementary file 1 — Supplementary Figures. [file 41598_2022_18950_MOESM1_ESM.pdf]

Supplementary

Tumor spheroids accelerate persistently invading cancer cells

Melanie Audoin[1,+], Maria Tangen Søgaard[1,2,+], and Liselotte Jauffred[1,*]

[1]The Niels Bohr Institute, University of Copenhagen, Blegdamsvej 17, DK-2100 Copenhagen O, Denmark
[2]Present address: DTU Health Tech, Denmark's Technical University, Ørsteds Pl. 344, 108, 2800 Kgs. Lyngby, Denmark
[*]jauffred@nbi.dk
[+]these authors contributed equally to this work

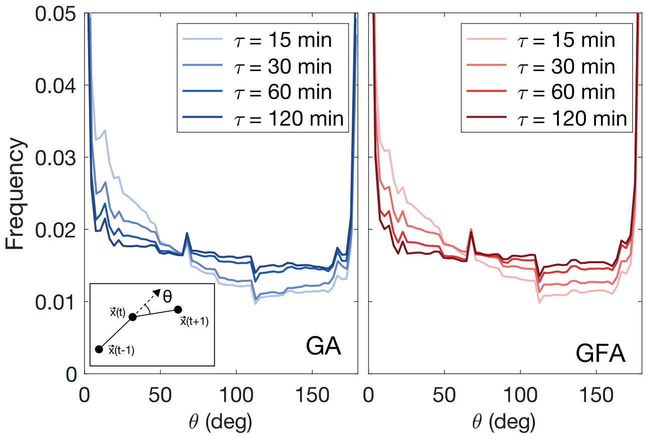

Fig. S1 Normalized and smoothed distribution of turning angles, θ, for GA (N=177) and GFA (N=305). The insert is a sketch of θ as defined in the main article. In both the GA and GFA cases, the distribution has local maximum at $\theta = 0$ and $\theta = 180°$and flattens for larger delays, τ.

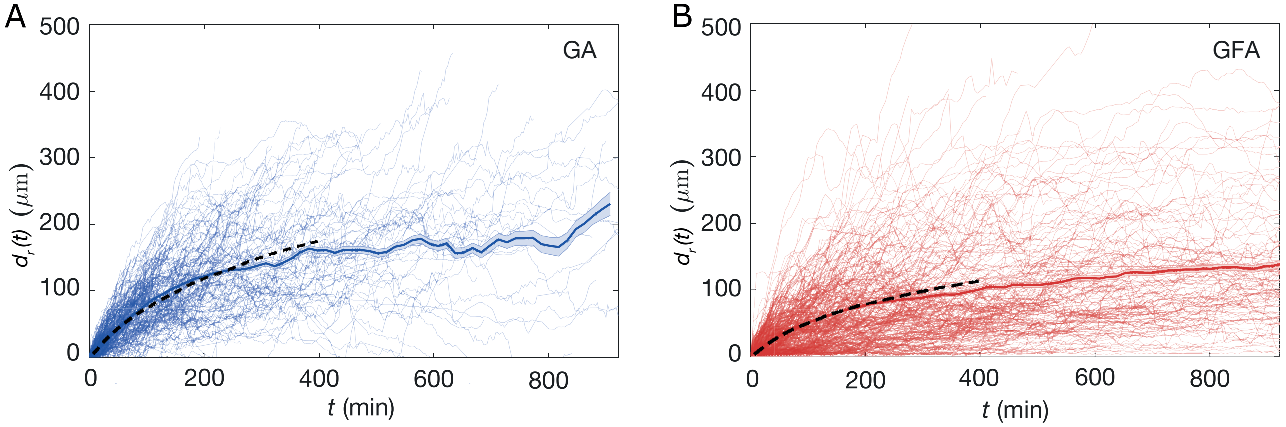

Fig. S2 Time evolution of the average migration displacement, $d(t)$, versus time. **(A)** The thin lines represents individual trajectories (N=177) and the full line is the average distance and shaded area is one SEM. The dotted black line corresponds to the average simulated trajectories using the BPRW model. **(B)** The thin lines represents all tracked trajectories (N=305) and the full line is the average distance and shaded area is one SEM. The dotted black line corresponds to the average simulated trajectories using the PRW model.

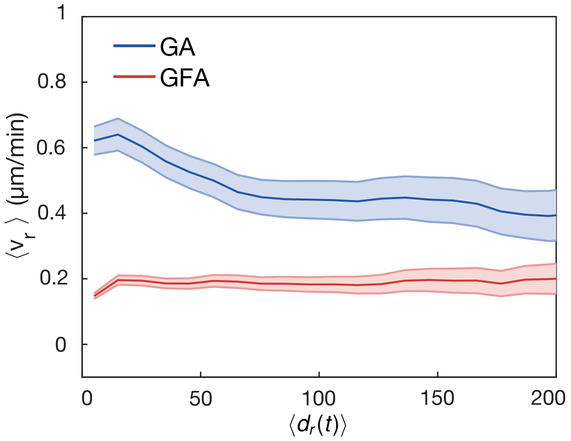

Fig. S3 Evolution of the ensemble-averaged radial velocity, $\langle v_r \rangle$, (mean ± SEM) against radial distance, $d_r(t)$ for both GA (blue) and GFA (red). v_r is binned on 15 µm with a moving average of 10 points.
